# Impact of reduced group size on patient adherence and functional outcomes in cardiac rehabilitation: insights from a COVID-19 pandemic natural experiment

**DOI:** 10.3389/fresc.2024.1465790

**Published:** 2024-09-11

**Authors:** Rachael M. Chait, Julia Ossi, Brett M. Colbert, Eric Huang, Juliann Gilchrist, Thais Garcia, Sharon Andrade-Bucknor, Azizi Seixas

**Affiliations:** ^1^ Miller School of Medicine, University of Miami, Miami, FL, United States; ^2^ Medical Scientist Training Program, Miller School of Medicine, University of Miami, Miami, FL, United States; ^3^ Department of Physical Therapy, Miller School of Medicine, University of Miami, Miami, FL, United States; ^4^ Department of Internal Medicine, Cardiology Division, Miller School of Medicine, University of Miami, Miami, FL, United States; ^5^ Department of Informatics and Health Data Science, Miller School of Medicine, University of Miami, Miami, FL, United States

**Keywords:** cardiac rehabilitation, 6-min walk test, patient-to-provider ratio, COVID-19, reduced group size

## Abstract

**Introduction:**

Cardiac rehabilitation (CR) adherence and functional outcomes were measured after COVID-19 regulations reduced group sizes to one-on-one, modeling a natural experiment.

**Methods:**

A retrospective analysis using a natural experiment model measured participants in 12 weeks of CR during the 17 months before and after a COVID-19-related closure was conducted. The age, sex, race, ethnicity, and referral diagnoses of the pre-COVID-19 closure and post-COVID-19 closure groups were analyzed using a student's unpaired T-test. Adherence (completion rate of CR) and functional outcomes [change in six-minute walk test (6MWT)] were assessed between the two groups using unpaired two-tailed student T tests in GraphPad Prism and confidence intervals were calculated with the Baptista-Pike method.

**Results:**

There were 204 patients in the pre-COVID-19 group and 51 patients in the post-COVID-19 group, due to the smaller group sizes in the post-COVID-19 group, with no significant differences in baseline characteristics between the groups. The pre-COVID-19 group had a higher patient-to-provider ratio [2.8 patients/provider (SD 0.74)] relative to the post-COVID-19 group [0.4 patients/provider (SD 0.12); *p *< 0.0001]. The post-COVID-19 group had a higher completion rate than pre-COVID-19 group [75% vs. 21%; OR 10.9 (95% CI, 5.3–21.3, *p *< 0.0001)]. Among those that completed CR, there was no significant difference between groups in 6MWT improvement [+377.9 ft. (*n* = 47; SD 275.67 ft.) vs. +346.9 ft. (*n* = 38; SD 196.27 ft.); *p *= 0.59].

**Discussion:**

The reduction in group size to one-on-one was associated with 10 times higher odds of CR completion. Among those that completed CR, functional outcomes were not influenced by group size. Thus, pursuit of one-on-one sessions may improve CR adherence.

## 1 Introduction

Cardiac rehabilitation (CR) is a comprehensive secondary prevention program with the goal of decreasing morbidity and mortality after a cardiac event. CR consists of three phases: acute inpatient therapy, outpatient, and maintenance (1). Phase 2 usually entails 36 guided exercise sessions over 12 weeks, with medically supervised aerobic and resistance exercises, nutritional counseling, and education about lifestyle modifications to manage cardiovascular risk factors. CR is known to have highly beneficial effects: reducing reinfarction and mortality after myocardial infarction (MI) by 47% and 36% respectively and decreasing total cholesterol, triglycerides, and systolic blood pressure, and increasing medication adherence (2–4).

Despite effectiveness of CR, use and completion rates remain low, approximately 20%–30% (5, 6). Several factors influence completion rates such as physician presence, patient-tailored programs, group solidarity, adequate space and equipment, fear of exercise, and team communication (7, 8). Currently, patients usually receive Phase 2 CR in group sessions, but the effect of group size on completion rates requires further study.

Having a sense of belonging and social identity can increase motivation and exercise adherence, aligning with the self-determination theory which states social relatedness is one of the three factors known to increase intrinsic motivation (7, 9, 10). Conversely, this study highlights how less participants per session allows for a more patient-tailored approach which can increase completion rates (11, 12). Thus, due to the natural reduction of group sizes following COVID-19 regulations, this study evaluates related CR completion and physiological outcomes compared to the previous mode of delivery.

## 2 Methods

### 2.1 Natural experiment model

A natural experiment is an observational research methodology where variation of the groups is not under the researchers' control (13). Classic examples of natural experiments are Jon Snow's cholera study in 1853 (14) and the use of twin studies (15). Specifically, the regression discontinuity design explores outcomes based on interventions with cut offs (i.e., age, geographic location, dates) (13). To control for confounding variables, this methodology uses a quasi-randomization technique, meaning that participants are still randomized while adhering to certain constrainsts (14). These experiments are highly utilized in public health research and can be more cost effective, improve internal and external validity, eliminate ethical considerations, and can influence more policy change compared to other methodology (13, 14, 16). This study leverages the natural experiment methodology by modeling the policy change during COVID-19 pandemic in the CR center to decrease group size, which accurately represents real-world change during this time.

### 2.2 Cardiac rehabilitation program

The cardiac rehabilitation program at the University of Miami is made up of 36 in-person sessions over 12 weeks. Patients are expected to attend two to three 60-min sessions per week. In each session, at least 40 min are spent on aerobic exercise and an average of 15 min on education. During a session, one therapist will be monitoring EKG readings while another therapist will be simultaneously working with a patient on exercises. Their progress is monitored by a physician who is the director of cardiac rehabilitation.

Prior to the COVID-19 pandemic, sessions were held in groups of 5–8 patients. After the onset of the COVID-19 pandemic due to social distancing guidelines, if two patients arrived during the same time slot, they did not work together, and the providers attended to them one-on-one.

### 2.3 Data collection

The CR program at the University of Miami Hospital was closed due to COVID-19 from March 16, 2020–May 2020. The 17 months prior to closure and 17 months after reopening were used as the cut off variation in the natural experimental model.

Prior to COVID-19, there were five sessions scheduled per day for 1.5 hours. Sessions were scheduled for six patients, although the average number of patients per session was three due to cancellations and no-shows. Post COVID-19 sessions were reduced to 1 hour to allow eight sessions per day. Due to space limitations, only two patients were scheduled per session with an average of one patient attending each session.

**Figure 1:**
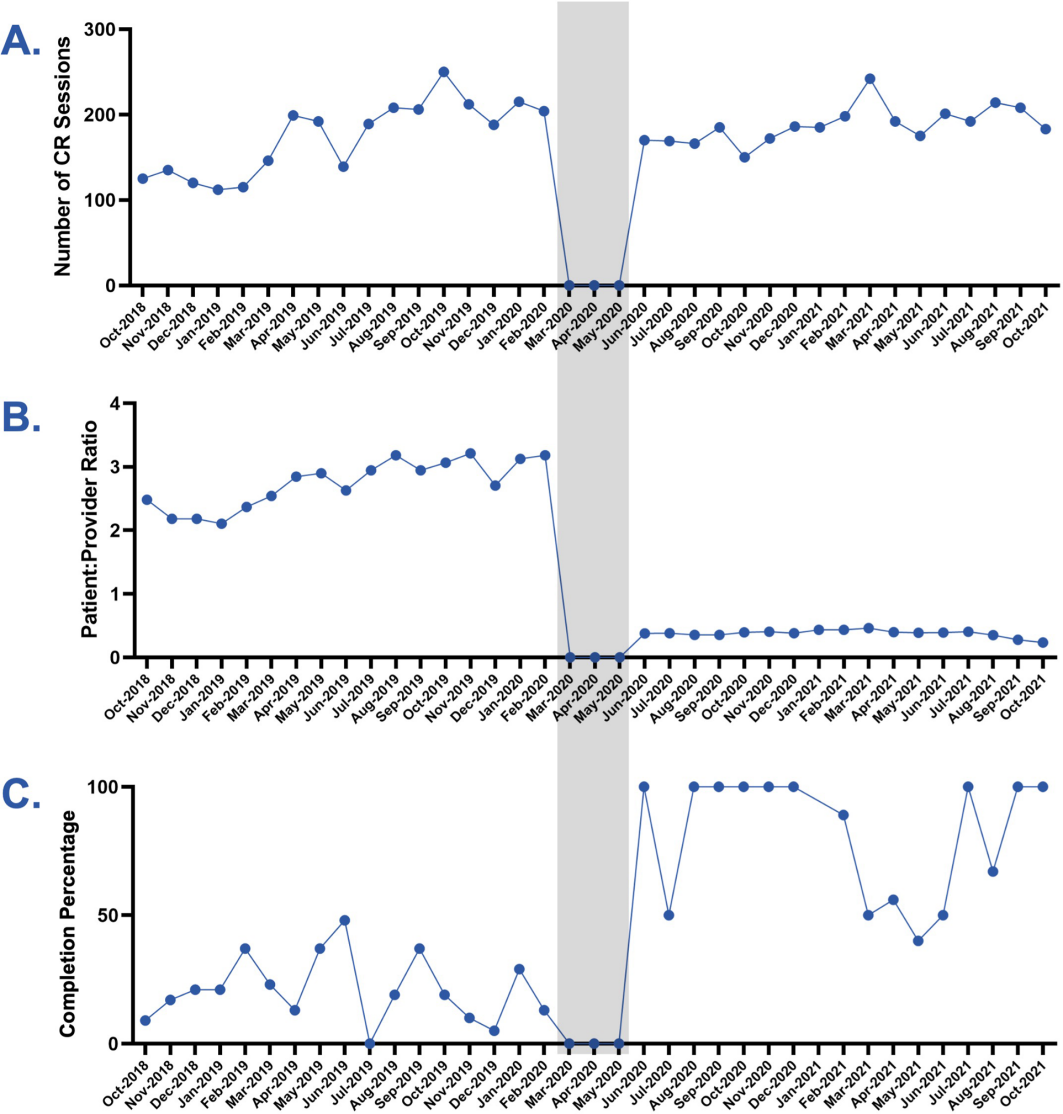
Charting findings before and after COVID-19. Line charts represent the 17 months pre and post-COVID-19. Gray shading indicates the time period of the 3 month facility closure. **(A)** Number of CR sessions by month. **(B)** Patient-to-provider ratio by month. **(C)** Completion percentage by month [graduates/(graduates + dropouts)].

**Figure 2:**
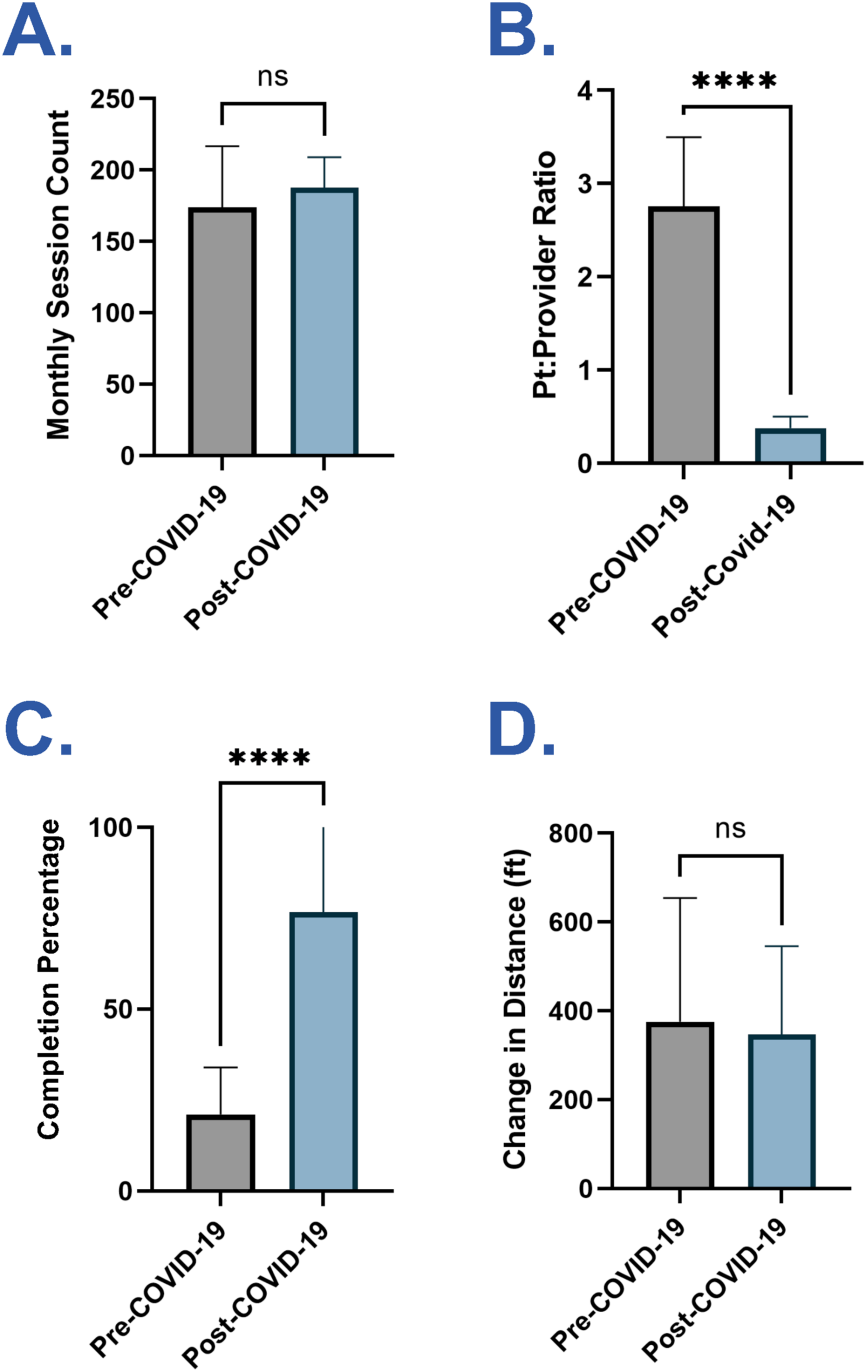
Comparison of mean outcomes before and after COVID-19. **(A)** Mean number of monthly CR sessions during the pre and post-COVID-19 periods. **(B)** Mean patient-to-provider ratio during the pre and post-COVID-19 periods. **(C)** Mean completion percentage during the pre and post-COVID-19 periods. **(D)** Mean change in 6MWT distance (ft) from baseline to graduation for graduates during the pre and post-COVID-19 periods. Data are presented as mean + SD. *p*-values were determined by unpaired, two-tailed student's *t*-test (ns, not significant; ****p < 0.0001; CR, Cardiac Rehabilitation; 6MWT, six-minute walk test).

Study approval was obtained from the University of Miami IRB (#20211122). Informed consent was not required as this was a retrospective, observational study. Participants were identified by chart review of patients who had intake sessions for Phase 2 CR during the time spans described above. There were 204 patients in the pre-COVID-19 group and 51 in the post-COVID-19 group due to policy change for COVID-19 regulations. Data on demographics, CR metrics, and completion rate was collected. Program information was also collected: the number of sessions per month (Figures 1A, 2A), the number of providers per session, and the number of patients per session.

### 2.4 Procedures

The patient-to-provider ratio was calculated by dividing the number of patients per session by the number of providers in session each day for the entire 17-month time span. Monthly means were calculated (Figure 1B). Mean patient-to-provider ratios were compared between the two cohorts (Figure 2B).

The dropout or completion status of each patient from Phase 2 CR was recorded. The monthly completion rate was calculated as the number of participants who completed CR divided by the sum of all participants who began CR each month (Figure 1C). Mean monthly completion rates were compared between the pre-COVID-19 and post-COVID-19 cohorts (Figure 2C).

The 6-min walk test (6MWT) is performed during the initial evaluation and discharge of each patient. It is a prognostic indicator recorded as the distance (feet) a patient can walk in six minutes, measuring the patient's functional capacity (17). The change in 6MWT distance between cohorts was calculated for each patient who completed Phase 2 CR. Mean scores were compared between the cohorts (Figure 2D). Change in 6MWT distance was only calculated for those that completed Phase 2 CR, as the test is only performed during intake (session 1 of 36) and discharge (session 36 of 36).

### 2.5 Statistical analysis

Descriptive statistics were used to compare baseline characteristics of the cohorts. Unpaired, two-tailed student's *t*-tests were used to compare pre- and post-COVID-19 means in GraphPad Prism, with alpha set at 0.05. Odds ratios were calculated, and confidence intervals were calculated using the Baptista-Pike method.

## 3 Results

### 3.1 Comparison of patient and institutional factors

Patient age, demographics, and referral diagnoses are comparable between the pre-COVID-19 and post-COVID-19 cohorts as there was no significant difference (Table 1). The average age in the pre-COVID-19 group was 65.6 (SD 13.6) years and in the post-COVID-19 was 65.0 (SD 12.35) years. The percentage of females in the pre-COVID-19 group was 32.4% and in the post-COVID-19 was 32.5%. Referral diagnoses were grouped by the categories listed in the CMS referral criteria (18) and percentages of each were comparable among cohorts (Table 1).

**Table 1:** Baseline characteristics.

	Pre-Covid-19 (*n* = 204)	Post-Covid-19 (*n* = 51)
Age, mean (SD), years	65.55 (13.63)	65.01 (12.35)
Sex, %		
Male	67.3	67.5
Female	32.4	32.5
Race, %		
Alaska Native/American Indian	0	0
Asian	2.9	2.1
Black	14.7	15.5
White	76.5	81.4
Native Hawaiian or Other Pacific Islander	0	0
Other	5.9	1
Ethnicity, %		
Hispanic	55.9	59.4
Non-Hispanic	44.1	40.6
Referring cardiac event, %		
MI/PCI	38.2	37.2
CABG	17.7	19.3
Stable angina	3.6	5.3
Valve repair/replacement	20.2	20.2
Heart transplant	0	0
Heart failure	20.3	18

Patients in the University of Miami's Cardiac Rehabilitation Program between October 2018-October 2021 were analyzed. There were no significant differences between the groups at baseline.

The number of sessions per month stayed constant while the number of patients decreased post-COVID-19. Except during the 3-month facility closure, the number of sessions held each month was comparable pre- and post-COVID-19 [173.8 (SD 41.7) vs. 187.5 (SD 20.82); *p *= 0.25; Figures 1A, 2A]. The number of patients was lower in the post-COVID-19 cohort (51 vs. 204) due to restrictions on the number of patients allowed in the facility.

The post-COVID-19 group had one-on-one sessions, unlike the pre-COVID-19 group. In addition to a lower number of patients scheduled per session after COVID-19, the CR facility added two more physical therapists during the sessions. The net result was a patient to provider ratio that was lower post-COVID-19 [0.4 patients/provider (SD 0.12)] compared to pre-COVID-19 [2.8 patients/provider (SD 0.74); *p *< 0.0001; Figures 1B, 2B] with the post-COVID-19 group often having one-on-one sessions. The ratios were steady in the 17 months before COVID-19 and the 17 months after COVID-19 (Figure 1B).

### 3.2 Comparison of outcomes

The change in 6MWT distance (ft) for discharges was comparable in the pre-COVID-19 and post-COVID-19 cohorts [+377.9 ft. (*n* = 47; SD 275.67 ft.) vs. + 346.9 ft. (*n* = 38; SD 196.27 ft.); *p *= 0.59; Figure 2D].

The completion rate was higher in the post-COVID-19 cohort than pre-COVID-19 cohort [75% vs. 21%; OR, 10.9 (95% CI, 5.3–21.3, *p *< 0.0001); Figures 1C, 2C]. The completion rate accounts for the proportion of patients who leave the program by completing it instead of dropping out early. This does not account for the total number of patients in the program at any given time, since each patient is at a different point in the 12 weeks of Phase 2 CR.

## 4 Discussion

Cardiac rehabilitation is an effective way to prevent morbidity and mortality after cardiac events (1–3) and operates in a dose-dependent manner (19). Yet, completion rates are low (approximately 20–30%), thus limiting the benefits of the program (6). Institutional factors (e.g., physician presence, adequate space and equipment, team communication, etc.) are known to influence completion rates, but there are mixed data on the effects of group size, specifically (7, 9, 10). This study uses a natural experiment model to evaluate how one-on-one group size sessions, with all other variables (e.g., session length, session activities, educational modules, etc.) remaining equal, affected completion rates. Though there was a large reduction in sample size, this was a unique opportunity to evaluate the effects of a natural reduction in group size and will help inform the new models of CR delivery on the rise, such as home-based CR.

The consistent decrease in patient-provider ratio from 2.8 patients/provider in the pre–COVID-19 group to 0.4 patients/provider in the post-COVID-19 group highlighted the change in class size. The pre-COVID-19 group had about six patients with one provider. This limited patient-provider direct interaction and patient-specific personalization, two factors known to influence completion rates (7, 20, 21). Post-COIVD-19, there were at most two patients with three providers in a session, with most sessions having only one patient. This allowed most patients to receive 12 weeks of one-on-one sessions that the providers could personalize to the needs of each patient. Having one-on-one attention could have also increased the patient's security in the effectiveness and understanding of CR. However, the smaller groups reduced the social cohesion typically seen in group exercise programs; this study measured if the positive effect of one-on-one sessions outweighed the negative effect of decreased social support on overall completion rates.

Pre-COVID-19, the completion rate at our center was 21%, similar to the national average (6). Post-COVID-19 closure, the completion rate rose to 75%, a significant increase (*p *< 0.0001; OR, 10.9; Figures 1C, 2C). To control for some possible confounders, analysis of the demographic profiles and referral diagnoses of the pre- and post-COVID-19 cohorts were performed and were equivalent (Table 1), suggesting an external factor accounted for the improved completion rate. One important external factor is the decreased patient-to-provider ratio, as all other aspects of the delivery of CR remained equal. Functional outcomes were measured by 6MWT, with equivalent improvements in the two cohorts for patients who completed CR (Figure 2D). The consistent improvement based on completion status suggests that at the individual level, completion is still the strongest predictor of a successful physiological response to CR, as noted by previous studies (2, 3, 19). However, at the population level, a higher completion rate means that a greater proportion of patients will experience these better outcomes.

This study was in a single CR center at a tertiary care hospital (University of Miami Hospital). The sample size differed largely between groups. However, while other factors remained constant, the natural experiment model allowed us to look at how one changed variable can affect adherence.

Barriers to access cardiac rehabilitation pre-pandemic included long driving times, parking costs, and lack of transportation (22–24). However, COVID-19 introduced variability out of the researchers' control such as patient risk category and mitigated pre-pandemic barriers. There was no access to information on patient socio-economic status or overall health. Patients had different risk tolerance to leave their home to attend CR in the post-COVID-19 group. Participants that are higher risk at baseline are more likely to adhere to and complete CR than lower risk patients (7, 20). However, given these limitations, there was baseline equivalence between groups. The effect size was also large, making it unlikely that the groups differed in such a way that would affect their outcomes.

Smaller group sizes may increase completion rates, however, this feasibility is limited by CR workforce availability. We propose implementing one-on-one sessions as a part of a greater CR model and suggest this as a future area of study.

The number of patients in each cohort was relatively small (pre-COVID-19 *n* = 204, post-COVID-19 *n* = 51), but it had diversity of age and referral diagnosis with a similar racial distribution to other large-scale CR studies (25). Though our study has limitations, we took advantage of a natural experiment model, and the effect size of the increase in completion rate was large (OR 10.9), suggesting a true effect.

## 5 Conclusion

Utilizing a natural experiment model showed that patients in one-on-one sessions had 10 times higher odds of adhering to the program. Group size had no effect on functional outcomes among those who completed CR (Figure 2D), reinforcing the importance of CR completion. As this was a natural observational study, there are many potential confounders. However, with such a large effect size, there is reason to consider adding one-on-one sessions to the potential models of CR delivery.

Another new model is home-based CR with a 34% decreased odds completing home-based CR compared to facility-based (20). Tailoring center-based rehabilitation to more one-on-one care or a hybrid model with home CR might improve completion rates and improve patient-centered care. Findings from this study can guide future research to where one-on-one sessions fit into the multiple modes of CR delivery, improving patient adherence and outcomes.

## Data Availability

The raw data supporting the conclusions of this article will be made available by the authors, without undue reservation. The regulatory steps to obtain de-identified data will be necessary and will include a data use agreement.
